# Successful treatment of methotrexate intolerance in juvenile idiopathic arthritis using eye movement desensitization and reprocessing – treatment protocol and preliminary results

**DOI:** 10.1186/s12969-018-0228-y

**Published:** 2018-02-13

**Authors:** Lea Höfel, Bruno Eppler, Magdalena Storf, Elizabeth Schnöbel-Müller, Johannes-Peter Haas, Boris Hügle

**Affiliations:** 1 German Center for Pediatric and Adolescent Rheumatology (DZKJR), Gehfeldstrasse 24, 82467 Garmisch-Partenkirchen, Germany; 2 Center for Pain Treatment in Young People, Garmisch-Partenkirchen, Germany

**Keywords:** Juvenile idiopathic arthritis, Methotrexate, Intolerance, Adverse effects, EMDR

## Abstract

**Background:**

Methotrexate (MTX), commonly used in juvenile idiopathic arthritis (JIA), frequently has to be discontinued due to intolerance with anticipatory and associative gastrointestinal adverse effects. Eye Movement Desensitization and Reprocessing (EMDR) is a psychological method where dysfunctional experiences and memories are reprocessed by recall combined with bilateral eye movements. The objective of this study was to assess efficacy of EMDR for treatment of MTX intolerance in JIA patients.

**Methods:**

We performed an open prospective study on consecutive JIA patients with MTX intolerance. Intolerance was determined using the Methotrexate Intolerance Severity Score (MISS) questionnaire prior to treatment, directly after treatment and after four months. Health-related quality of life was determined using the PedsQL prior to and four months after treatment. Patients were treated according to an institutional EMDR protocol with 8 sessions over two weeks. Changes in MISS and PedsQL were analyzed using non-parametric statistics.

**Results:**

Eighteen patients with MTX intolerance (median MISS at inclusion 16.5, IQR = 11.75–20.25) were included. Directly after treatment, MTX intolerance symptoms were significantly improved (median MISS 1 (IQR = 0–2). After four months, median MISS score was at 6.5 (IQR = 2.75–12.25, *p* = 0.001), with 9/18 patients showing MISS scores ≥6.

Median PedsQL after 4 months improved significantly from 77.6% to 85.3% (*p* = 0.008).

**Conclusion:**

MTX intolerance in children with JIA was effectively treated using an EMDR protocol, with lasting effect over a period of 4 months. EMDR treatment can potentially increase quality of life of affected patients and enable continued MTX treatment.

**Electronic supplementary material:**

The online version of this article (10.1186/s12969-018-0228-y) contains supplementary material, which is available to authorized users.

## Background

Methotrexate (MTX) is the most commonly used medication in the treatment of children with inflammatory joint diseases, especially for polyarticular juvenile idiopathic arthritis (JIA) [1, 2]. Treatment with low-dose MTX therapy exhibits few serious adverse effects; treatment is usually discontinued due to intolerance symptoms such as anticipatory nausea and refusal to take the medication [3, 4]. The origin of MTX intolerance is still unclear, and common countermeasures have been shown to be ineffective in suppressing MTX intolerance [5, 6]. Current studies demonstrate high rates of intolerance for JIA patients on treatment with low-dose MTX, while high-dose regimens e.g. in the treatment of childhood cancer, are usually much more responsive to medication with antiemetics [6–9].

EMDR (Eye Movement Desensitization and Reprocessing) is a psychological method that has historically been applied to the treatment of posttraumatic stress disorder, but has since then been developed into an overall approach for other adverse life experiences. The eight-phase treatment approach is composed of standardized protocols and procedures, using intensive recall of unprocessed memories while applying bilateral eye movements, taps, or tones [10]. As a result, affective distress is relieved, negative beliefs are reformulated, and physiological arousal is reduced.

The efficacy of EMDR for trauma-related disorders has been shown in controlled trials for the treatment of trauma in children and adolescents, but also in other fields of stress related psychological disorders, such as post-operative pain or seizure-related post-traumatic stress [11–13].

While EMDR has not been used for the treatment of MTX intolerance so far, a treatment approach would be based on the hypothesis that MTX treatment in JIA patients is a repetitive stressful or even traumatic event leading to anticipatory and associative adverse effects that prevent healthy information processing. The objective of this study was to describe the EMDR protocol for the treatment of MTX intolerance, as well as to determine efficacy of EMDR when used to treat MTX intolerance in children with JIA on continued MTX treatment.

## Methods

### Patients

Consecutive patients admitted to the German Center for Pediatric and Adolescent Rheumatology from October 2016 until May 2017 for planned EMDR treatment were included in this study. Inclusion criteria were 1) diagnosis of JIA according to ILAR criteria [14], 2) age between 8 and 17 years, 3) symptoms of MTX intolerance, as determined by questionnaire (see below), and 4) necessity of MTX treatment for at least 6 more months as determined by the treating physician. Intolerance to MTX was determined using the Methotrexate Intolerance Severity Score (MISS) questionnaire, previously developed and validated in JIA [7]. The MISS consists of four domains: abdominal pain, nausea, vomiting and behavioral symptoms, assessing symptoms after MTX administration, anticipatory and associative symptoms. The behavioral symptoms domain includes restlessness, irritability and refusal of MTX, which develop in response to MTX-induced gastrointestinal symptoms and anticipation thereof. A patient could score 0 (no symptoms), 1 (mild symptoms), 2 (moderate symptoms) or 3 (severe symptoms) points on each item. MTX intolerance was defined as ≥6 points, including at least one anticipatory, associative or behavioral symptom [3]. Exclusion criteria were 1) other diseases leading to nausea and/or abdominal complaints, 2) concomitant medications possibly inducing nausea (excepting biologics and non-steroidal anti-inflammatory drugs) and 3) pre-existing contraindications that would prevent EMDR treatment such as dissociative disorders, personality disorders or severe somatic disorders (e.g. cardiac arrhythmias).

Written consent was obtained from the parents prior to inclusion in the study. The study was approved by the ethics committee of the Medical Faculty of the Ludwig-Maximilian University, Munich, Germany.

### Intervention

Treatment was performed by two pediatric psychologists (BE and MS) trained in EMDR technique following an institutional EMDR protocol. The standard EMDR protocol, which includes the 8 phases “history taking”, “preparation”, “assessment”, “desensitization”, “installation”, “body scan”, “closure” and “reevaluation” was adapted for the treatment of MTX intolerance [15]. The protocol is described in detail in Additional file 1: Table S1.

Treatment started with an initial session consisting of a structured psychosocial and medical history, including preexisting traumatic experiences and the development of the MTX intolerance reaction as well as education about the method. At the end of the first session, a ‘calm place’ exercise was conducted in order to address potential paradoxical anxiety responses, using a slow bilateral stimulation while the patients envisioned a place where they felt calm and peaceful (phases 1 and 2 of the protocol).

Subsequently, five sessions lasting 60 min each were held over a time period of 10–12 days according to the standard EMDR protocol (phases 3–8 of the protocol), concluding with the forthcoming and thus anticipated MTX application.

The final two sessions consisted of the application of Methotrexate, without supervision of the therapist, and a final session following the MTX application, where the positive experiences were installed and a possible “worst case” scenario in the future was reprocessed to lessen residual anticipatory anxiety or avoidance (future template). Parents were present at the first session, during the application of MTX and for the closing meeting.

### Data acquisition and analysis

Primary outcome was improvement of MTX intolerance measured by MISS 4 months after therapy. MISS was measured prior to the first therapy session, directly after the last therapy session and 4 months later [7]. Health-related quality of life was also measured prior to therapy and 4 months after therapy, using the PedsQL 4.0 Generic Core Scales [16]. The following data was extracted from patient files: age, gender, body weight and height (to calculate methotrexate dose per body surface area), age at diagnosis, duration of disease, methotrexate dose, route of administration and folic acid supplementation and were analyzed using descriptive statistics. MISS score changes over time and changes of PedsQL over time were compared using non-parametric Wilcoxon Signed Rank tests. Statistical analysis was performed using SPSS version 21.0 (SPSS Inc., Chicago, IL, USA).

## Results

Eighteen patients were included in the study. Demographic and clinical data of the patients are shown in Table 1. Patients did not change route or dose of MTX administration during the study.

**Table 1:** Baseline demographic data of patient cohort

Characteristics	*N* = 18
Gender, female	16 (89%)
Age, mean ± SD	13,9 ± 2.9 years
JIA subtype	
Oligoarticular, persistent	5 (28%)
Oligoarticular, extended	6 (33%)
Polyarticular, rheumatoid-factor negative	4 (22%)
Psoriatic arthritis	2 (11%)
Enthesitis-associated arthritis	1 (6%)
Disease characteristics	
ANA positive	14/18 (78%)
HLA-B27 positive	4/18 (22%)
Age at diagnosis, mean ± SD	6.8 ± 4.4 years
Disease duration, mean ± SD	7.1 ± 4.1 years
Medications	
MTX route of administration, subcutaneous	11/18 (61%)
MTX duration use, mean ± SD	59 ± 40 months
MTX dose, mean ± SD	12.3 ± 1.6 mg/sqm/week
Additional medication	
TNFα inhibitors	7/18 (39%)
NSAIDs, regularly	2/18 (11%)
Folic acid	16/18 (89%)

### Methotrexate intolerance and change of MISS score over time

**Fig. 1:**
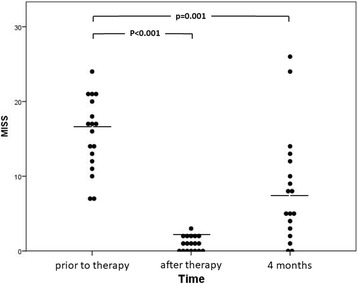
MISS scores of the cohort prior to, directly after and four months after EMDR treatment

All patients showed MTX intolerance prior to treatment (defined as MISS score ≥ 6), with a median MISS score of 16.5 (IQR = 11.75–20.25, mean: 15.6). Directly after EMDR treatment, median MISS score decreased significantly to 1 (IQR = 0–2, mean: 1.0, *p* < 0.001). No patient had MISS scores ≥6 at this time, and all reported subjective improvement compared to prior to EMDR treatment when MTX medication was applied. 4 months after treatment, median MISS score was at 6.5 (IQR = 2.75–12.25, mean: 8.3, *p* = 0.001) (Fig. 1). 9/18 patients (50%) showed MISS scores ≥6 at this time.

### Quality of life and change of PedsQL score over time

**Fig. 2:**
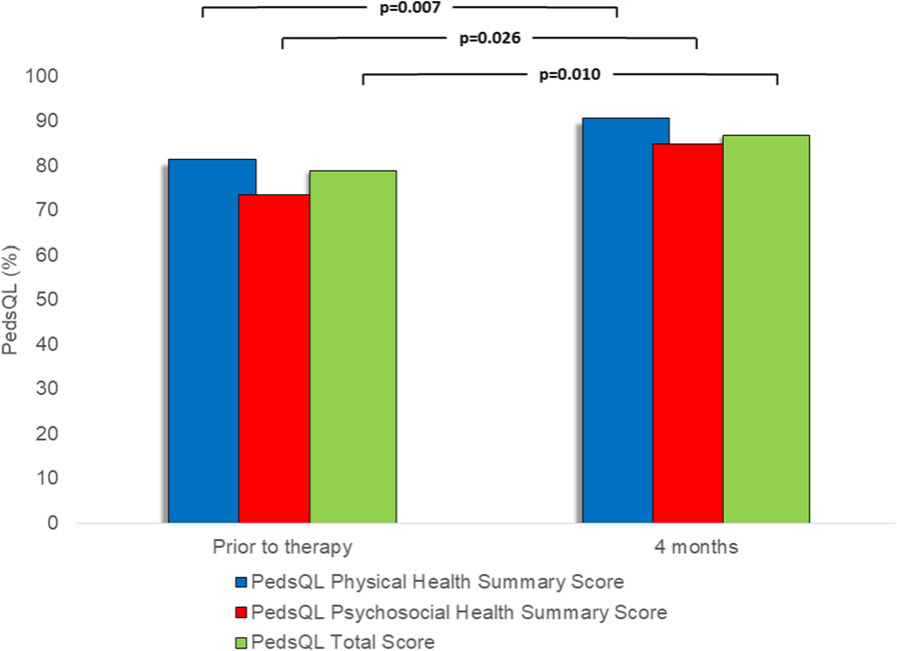
PedsQL scores of the cohort prior to, and four months after EMDR treatment

Median health related quality of life as measured by the PedsQL questionnaire was 77.6% (range 46.7% – 100%) prior to treatment. 4 months after treatment, PedsQL was significantly improved with a median PedsQL score of 85.3% (range 64.1% – 100%, *p* = 0.008) (Fig. 2). The median Physical Health Summary Score of the PedsQL improved from 76.3% (range 40.6% – 100%) to 82.7% (range 59.4% – 100%, *p* = 0.026), while the median Psychosocial Health Summary Score of the PedsQL improved from 77.3% (range 37.5% – 100%) to 86.4% (range 59.4% – 100%, *p* = 0.007).

## Discussion

Methotrexate is a commonly used and highly effective drug in the treatment of children with rheumatic diseases, with very limited adverse effects. Discontinuation of an obviously effective and, besides the intolerance, well-tolerated medication can be extremely frustrating for patients, parents and care providers alike. In this study we demonstrate that treatment with EMDR has the potential to significantly alleviate MTX intolerance symptoms, with a sustained effect over 4 months. We were also able to show that quality of life in treated patients had improved after 4 months, with the largest improvement found in the physical health subscore of the PedsQL. This either results from MTX intolerance being experienced primarily as a physical phenomenon by the affected patients or, EMDR treatment having a more pronounced effect on the physical sensations of MTX intolerance.

These preliminary results indicate that EMDR treatment has had a beneficial effect on patients with MTX intolerance; besides improving the quality of life, this treatment also has the potential to prevent medication changes which do not only carry increased risk for the patients, but frequently lead to the incurrence of substantial costs to the families and the coverage provider alike.

The concept of EMDR is based on the Adaptive Information Processing Method, stating that, when experiences are processed in a healthy way, multiple elements of the experience such as thoughts, images, emotions and sensations are stored in our memory and helpful associations are forged with stored experiences and reactions in memory. If a disturbing or traumatic event occurs, this information processing may not be complete. Strong feelings or dysfunctional coping mechanisms may interfere with information processing. This interference then prevents the access to more adaptive and healthy information that is stored in our memory networks. One might be stuck in the experience loop of a visual flashback or an emotion without being able to adapt and learn from it. The experience is then unprocessed and the physiological system is not able to return to a healthy, stable baseline – the emotions are not in balance.

EMDR enables the processing of dysfunctional and traumatic memories, using an intensive recall combined with bilateral stimulation usually evoked by eye movement to dissolve the memories by reprocessing them. As a result, affective distress is relieved, negative beliefs are reformulated, and physiological arousal is reduced. Unprocessed memory content and dysfunctional experiences and memories are reprocessed several times in order to enable healthy information processing [15]. The traumatic memory is desensitized by short imaginal exposure to this memory and subsequent bilateral stimulation, which is repeated until the accompanying subjective sense of disturbance has disappeared. Bilateral stimulation by eye movement is supposed to counteract the ‚frozen information‘, enabling dissolution of traumatic memories, neutralization of the negative affect and reduction of physiological arousal [10].

EMDR has proven an effective treatment of traumatic stress disorder in adults and children [17–20]. Whilst EMDR is most frequently used in the treatment of trauma- and stress-related disorders, its usefulness has been demonstrated in the treatment of other kinds of dysfunctional memories and inefficient information processing of further adverse life experiences [11, 12].

For this study, we assumed that MTX intolerance is based on dysfunctional or incomplete information processing evoked by e.g. strong negative feelings or adverse anticipation of side effects that can be considered similar to a trauma- or stressor-related disorder. This is in keeping with the adaptive information processing model of EMDR, which states that dysfunctionally stored and not fully processed memories are the cause of various mental disorders, or more broadly “that after a certain event, a certain psychopathology appears, which can be effectively addressed by EMDR therapy” [21]. Being diagnosed with a rheumatic disorder and being treated with MTX might not necessarily qualify as severe mental or emotional stress or physical injury alone [22]. However, patients have experienced MTX-related adverse effects or might have been told of the possibility of these occurring, and thus anticipate them in the future, with regular weekly re-enforcements and virtually no chance for escape. The behavioral distress such as showing panic or resistance at the sight of the medication frequently reported in children with MTX intolerance fits well within the symptom clusters of trauma- and stress related disorders [4, 7].

While patients improved significantly directly after EMDR treatment, a significant number showed symptoms of MTX intolerance at follow-up 4 months later. Continuous treatment with MTX could arguably re-initiate the same process that led to the intolerance symptoms in the first place; however, in most cases the EMDR treatment, including techniques imparted on the affected patients, seem to provide a measure of protection. A possibly neglected factor is the education of the parents; personal communications in selected cases suggested an influence of parental expectations on recurrence of MTX intolerance symptoms. Possible improvements to the EMDR protocol in the future include one or several regularly scheduled follow-up treatment sessions as well as an in-depth education of the parents included in the primary protocol.

MTX intolerance as a unified construct of the anticipatory and associative side effects associated with prolonged use of MTX has only recently been described, and the MISS questionnaire tries to capture this construct [7]. So far, genetic studies have been unsuccessful in finding a causal link to MTX intolerance [5, 23, 24]. Interaction between the patient and his parents, physicians and other caregivers as well as psychological and social background factors can all influence frequency and severity of MTX intolerance symptoms, and therefore the efficacy of any intervention. A limitation of this study was patient selection, including only patients with sufficiently severe symptoms of MTX intolerance to be willing to undergo two weeks of (partially in-patient) treatment. This was not a randomized trial but a mere ‘proof of concept’, and there was no control group with ‘treatment as usual’; however, it has previously been shown that untreated or conventionally treated MTX intolerance tends to get worse over time rather than improve on its own [6]. The significant benefits the patient received from the treatment in this study argues for the efficacy of the EMDR approach in the treatment of MTX intolerance. Further studies are necessary to elucidate not only the cause of MTX intolerance, but also the exact benefits of EMDR treatment for MTX intolerance.

## Conclusion

Patients with JIA showing MTX intolerance profited significantly from EMDR treatment directly after the treatment and over a period of 4 months, allowing continuation of MTX treatment with improved quality of life. To our knowledge, this is the first report of an efficacious measure against MTX intolerance, which is developing into one of the largest problems in the day-to-day treatment of JIA patients in the clinic. Further studies should investigate long-term efficacy of this treatment approach, if re-treatment is necessary, and if yes, in what format.

## Additional file

Additional file 1: **Table S1.** EMDR standard protocol for MTX intolerance. (DOCX 37 kb)
