# Supplementary material for: Successful treatment of methotrexate intolerance in juvenile idiopathic arthritis using eye movement desensitization and reprocessing – treatment protocol and preliminary results
Source: Pediatr Rheumatol Online J. 2018 Feb 13;16:11. doi: 10.1186/s12969-018-0228-y (PMC5809965; doi:10.1186/s12969-018-0228-y)
Supplement: Supplementary file 1 — Table S1. EMDR standard protocol for MTX intolerance. (DOCX 37 kb) [file 12969_2018_228_MOESM1_ESM.docx]

| **Protocol for Treatment of MTX intolerance using EMDR^[[1]](#footnote-1)^**  ***Eppler B., Storf L., Höfel L., Hügle B., Schnöbel-Müller E., Haas J.P.***  ***German Center for Pediatric and Adolescent Rheumatology (GCPAR)***  ***Center for Pain Treatment in Young Persons*** | | |
| --- | --- | --- |
| **1.** |  | **Phase 1 + 2 – patient together with parent(s)** |
| **Taking of History** | Evaluation of somatic and psychological pre-existing stressors/conditions including history of previous trauma.  Detailed history of development of MTX intolerance.  Assessment of time course of MTX intolerance reaction:   - Start of negative reaction prior to MTX application? - Duration of the negative reaction? - Changes of the negative reaction over its course?   Check for contraindications:   - Severe dissociative disorder - Personality disorders - Seizure disorders - Severe and/or life-threatening somatic disorders (e.g. cardiac arrhythmias, cardiac arrest, anaphylactic shock) |  |
| **2.** |  |  |
| **Education on EMDR** | The patient is informed that treatment with MTX is interrupted until he/she is ready to recommence and is confident to tolerate the treatment.  ‘Frozen’ memory content that is stored in a dysfunctional manner, consisting of scenic memories, concomitant emotions, cognitions and physical sensations will be reprocessed to decouple the explicit, event-related memory from accompanying emotions and physical sensations. Remembering the situation will then not automatically cause emotions and physical sensations similar to the original experience.  Consent is obtained from the patient. |  |
| **3.** |  |  |
| **Calm place exercise with EMDR** | Positive recollections from the patient memory together with accompanying emotions and physical sensations are actively recalled.  Seating position, distance, hand spacing, direction and technique for bilateral stimulation by eye movement or alternative tapping are explained and practiced.  Interruptions for feedback and stop signals are established. The patient can always interrupt the session.  Memory + emotion + physical sensation are activated and installed as a resource with slow, bilateral stimulation. Besides activating resources this familiarizes the patient with the EMDR method in a positive setting. |  |

| **4.** |  | **Phase 3** |
| --- | --- | --- |
| **Target** | Repetition of the calm place exercise (see point 3) is optional.  Physical and psychological well-being is determined.  Only when little or no complaints are reported, patient and therapist proceed to the next step.  Target for treatment of MTX intolerance is established (original experience):   - *“If you think of MTX* ***now****, which memories, emotions, thoughts and images appear in your mind?”* - *“Which of these cause you the most discomfort? “*   Target can relate to a specific instance of MTX application, e.g. the worst moment.  Target can also generally refer to nausea, color, or other circumstances of MTX application (e.g. being held down by caregivers, smell of disinfectant, click of the applicator pen etc.) |  |
| **5.** |  |  |
| **NC**  Negative Cognition | Negative Cognition (NC)  *- “Which negative thoughts are triggered by this?”*  *- “What do you believe of yourself or of MTX* ***now****?”*  (e.g. I cannot tolerate that; I always feel nauseated by that; I don’t want that; my body cannot sustain that etc.) |  |
| **6.** |  |  |
| **PC**  Positive Cognition | Positive Cognition (PC)  *- “What would you like to believe about yourself* ***now****?”*  (e.g. the medication helps against my rheumatic disease; I tolerate the medication well; I will tolerate the drug as well as other drugs; it hurts for a brief period of time but I can manage this well etc.) |  |
| **7.** |  |  |
| **VoC** | VoC: Validity of Cognition    *“When you focus on that image / that incident and think of those words* (therapist repeats PC as an “I” statement)*, how true do these words feel to you* ***now****? “*  1 2 3 4 5 6 7  (completely false) (completely true) |  |
| **8.** |  |  |
| **Emotions** | Emotions/feelings  *“If you focus on that image / that incident and think of those words* (therapist repeats NC as an “I” statement)*, which emotions do you feel* ***now****?“* |  |

| **9.** |  |  |
| --- | --- | --- |
| **SUD** | SUD: Subjective Units of Disturbance  *“How disturbing does the incident feel to you* ***now*** *on a scale from zero to ten?“*  0 1 2 3 4 5 6 7 8 9 10  (no disturbance or neutral) (highest disturbance)  If no disturbance is reported by the memory, the SUD can determine the extent of nausea (0 – no nausea; 10 – maximal nausea). |  |
| **10.** |  |  |
| **Location of Body Sensation** | Body Location  Localization and description of physical sensations when envisioning the target, the NC (see point 5) and the emotions.  *“Where do you feel the disturbance in your body most* ***now****? How does that feel?“* |  |

| **11.** |  | **Phase 4** |
| --- | --- | --- |
| **Desensitization** | Desensitization/reprocessing with the target, NC (see point 5), emotion (see point 8) and body sensation (see point 10) with fast bilateral stimulation.  *“Please envision the worst moment*  ***now*** (therapist recaps the situation) *and remember the negative sentence* (therapist repeats NC as an “I” statement) *and notice your feelings* (name feelings) *and what you feel in your body* (name physical sensations) *“*  *“Observe what happens within you and follow my finger.”*  Interrupt after a set of bilateral provocations:  “*What do you notice* ***now****?”*  Continue after one or two cues or sentences by the patient, until neutral or positive content surfaces at least twice.  *“Return to the original experience* (name target as a heading)*. What do you notice* ***now****? How disturbing does it feel to you on a scale from 0 – 10* (SUD value)*?”*  0 1 2 3 4 5 6 7 8 9 10  (no disturbance or neutral) (highest disturbance)  If disturbance is still present:  *“What causes the disturbance to still be at xy* (SUD value)*?”*  Reprocess until SUD = 0. (In case of persisting disturbance, return to calm place exercise and/or stabilizing measures, e.g. container exercise.) |  |
|  | | |

| **12.** |  | **Phase 5** |
| --- | --- | --- |
| **Installation** | In case of complete reprocessing:  *“Please remember the original experience; is the good sentence (*therapist repeats PC as an “I” statement) *still correct, or is there a better one?”*  *“When you focus on the original experience and think of those words, how true do these words feel to you* ***now*** *on a scale from 1 to 7?*  1 2 3 4 5 6 7  (completely false) (completely true)  Install with slow bilateral stimulation (repeat until VoC ≥6) |  |
|  | | |
| **13.** |  | **Phase 6** |
| **Body Scan** | Body Scan  *“Please close your eyes and hold in mind the original experience and those words* (repeat the selected PC)*. How does that feel?”*  If discomfort is felt, this can be either immediately reprocessed or the patient can be distanced from it (calm place exercise or container exercise, as needed) |  |
|  | | |
| **14.** |  | **Phase 7** |
| **Closure** | Closure of the session  Feedback about the process: positive reinforcement if patient has stayed close to the target over the whole session.  Explain continuation of processing.  *“The processing we have done today may continue after the session, e.g. by dreams, thoughts or memories. Just let this happen, we can work on this in the next session.”* |  |
|  | | |
| **15.** |  | **Phase 8** |
| **Reevaluation** | Reevaluation  If at the start of the next session the patient reports disturbance by the original experience and/or other stressful memories resurface, the protocol should be repeated using the appropriate target (points 1- 14)  If at the start of the next session no disturbance by the original experience is reported, the forthcoming (anticipated) MTX application is established as the new target (future template). Follow the protocol with this target.  Continue until no disturbance is reported. |  |

**Overview of the procedure**

- Diagnosis of MTX intolerance
  - Medical history
  - Objective diagnostic using the Methotrexate intolerance severity score (MISS), pathologic score ≥ 6
- Protocol
- MTX application in-vivo
- Installation of the positive MTX experience with slow bilateral stimulation
- Installation of the “worst case” future template

**Additional interventions for blocking beliefs / rumination**

- Imaginative resources and body resources (e.g. Antidote)
- Distancing and change in perspective (e.g. cinema technique)
- Externalisation (e.g. image presentation)
- Affect bridge (work with background experiences)

1. Eye movement desensitization and reprocessing [↑](#footnote-ref-1)
